# Amber-red color imaging makes the dissection line more evident during gastric endoscopic submucosal dissection

**DOI:** 10.1055/a-2694-7445

**Published:** 2025-09-15

**Authors:** Kohei Funasaka, Ryoji Miyahara, Noriyuki Horiguchi, Hyuga Yamada, Keishi Koyama, Gakushi Komura, Seiya Hagihara, Hijiri Sugiyama, Mizuki Ariga, Mitsuo Nagasaka, Eizaburo Ohno, Teiji Kuzuya, Yoshiki Hirooka

**Affiliations:** 1 12695 Department of Gastroenterology and Hepatology, Fujita Health University, Toyoake, Japan

**Keywords:** Endoscopy Upper GI Tract, Endoscopic resection (ESD, EMRc, ...), Quality and logistical aspects, Image and data processing, documentatiton, Performance and complications

## Abstract

**Background and study aims:**

Local injection of a small amount of blue dye into the submucosa can facilitate recognizing the dissection line in endoscopic submucosal dissection (ESD). Amber-red color imaging (ACI), which hardly affects the submucosal blue color, is suitable for the entire ESD. This study aimed to clarify characteristics of ACI during ESD.

**Patients and methods:**

Nine endoscopic images were selected during submucosal dissection in four cases of gastric ESD to evaluate endoscopic ACI and white light imaging (WLI). Visibility of the dissection line and the submucosal vessel were evaluated by eight endoscopists using a 5-point Likert scale. The blue submucosal area of each endoscopic image and color signal surrounding the submucosa were compared between ACI and WLI. In addition, the color signals in gradient dilutions of blue solutions were compared in ex vivo experiments.

**Results:**

Visibility of the dissection line was better in ACI than in WLI and visibility of the submucosal vessels was slightly better in ACI. The size ratio of the blue area in ACI and WLI (i.e., ACI/WLI) ranged from 0.53 to 0.65, indicating that the blue area in the ACI was narrower. The red signal intensity of the surroundings with respect to the submucosa was greater in ACI than in WLI, which was related to the narrower blue area in ACI. Ex vivo experiments corroborated this observation.

**Conclusions:**

ACI highlights the submucosa in blue only where sufficient solution is injected, which facilitates recognition of the dissection line during ESD.

## Introduction

Endoscopic submucosal dissection (ESD) has been established as a standard therapy for early neoplasms in the gastrointestinal tract and is widely used throughout Japan and worldwide 1 2 3 4 5 6 . Details of ESD techniques differ depending on the device used, but the fundamental steps of ESD are consistent 7 8 9 . In particular, injecting a solution into the submucosal layer is essential for distinguishing the mucosal and muscular layers to allow incision and dissection without damage or perforation of the muscular layer.

A small amount of indigo carmine in solution has been injected empirically since the 1980s, before ESD. Typically, once a solution (e.g., glycerol or hyaluronic acid mixed with indigo carmine) is injected into the submucosal layer, the mucosa is elevated, and the submucosa becomes thick and is colored light blue. The endoscopist can easily recognize the submucosa as a blue area and dissect the submucosa directly without muscular damage. If the injected solution is not blue, the appropriate dissection line is difficult to recognize, which can cause muscular damage or perforation.

Red dichromatic imaging (RDI) was developed as a novel form of image-enhanced endoscopy (IEE) that uses two long-wavelength (600 nm and 630 nm) lights provided by Olympus Co. (Tokyo, Japan), which was named dual-red imaging in the past. This IEE visualizes thick vessels in the deeper mucosa or submucosa 10 and improves visibility of the bleeding points in ESD 11 12 13 . However, the color of the submucosa injected with blue dye looks deep purple. Because it is difficult to evaluate the depth of the submucosa in RDI, the submucosa has to be dissected carefully. In a clinical study, RDI was applied only in bleeding situations in ESD 14 . Currently, ESD is still performed using white light imaging (WLI) with the exception of hemostasis.

In this context, to improve the color tone of the submucosa, we evaluated a new image enhancement technology using white light illumination developed by FUJIFILM Co. (Tokyo, Japan) 15 . By further improving this technology and modifying the illumination light, FUJIFILM has recently developed a novel IEE called amber-red color imaging (ACI). ACI improves visualization of blood flow in bleeding situations through use of amber and orange colors. In addition, ACI maintains other color tones, especially blue tones, similar to WLI. Thus, ACI is suitable for the entire ESD 16 . In our experience of gastric ESD using ACI, the blue contrast between the submucosa and the surrounding layers is more obvious in ACI than in WLI, and the dissection line was quickly recognized in ACI. Therefore, we clarified characteristics of ACI during ESD by comparing endoscopic images and optical characteristics obtained using ACI with those of WLI.

## Patients and methods

### Patients

**Fig. 1:**
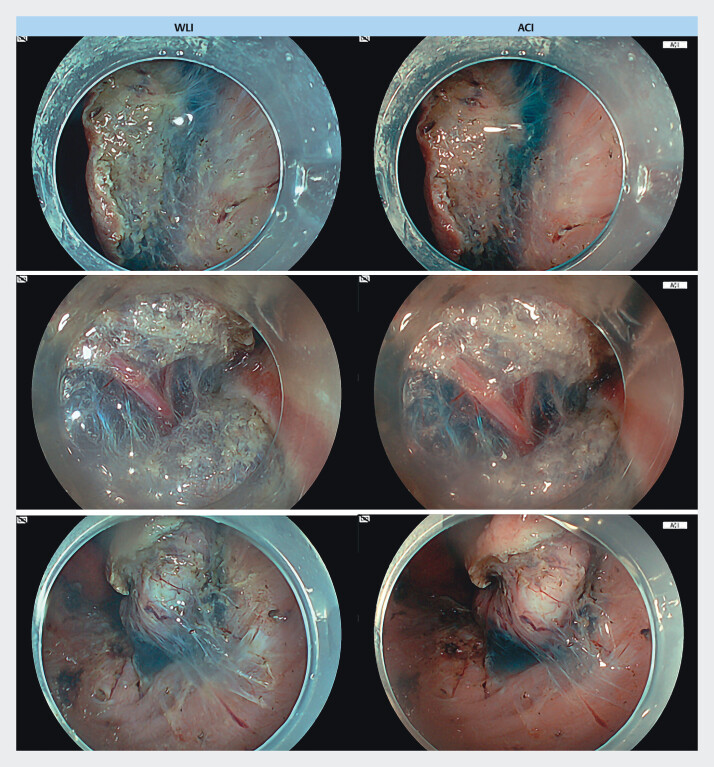
Representative endoscopic image of submucosal dissection using WLI (left) and ACI (right).

We evaluated four gastric ESDs performed with an endoscope EG-840T and a processor EP-8000 (FUJIFILM Co., Tokyo, Japan) from August to September 2024 at Fujita Health University Hospital. These ESDs were performed by one endoscopist (F.K.) with experience in more than 500 ESDs. After whole circumferential marking, ESD was performed using ACI. Endoscopic still images with ACI and WLI were captured in the same images during submucosal dissection ( Fig. 1 ). The concentration of indigo carmine in the solution for submucosal injection was set at 0.5%. The Ethics Committee of Fujita University approved this retrospective study (IRB No. HM24–147). Patients could withdraw from the investigation via the opt-out method provided on the hospital website.

### Characteristics of ACI

ACI is characterized by use of amber-red, green, and blue LEDs ( **Supplementary Fig. 1a** ). The aim of ACI is to support identification of bleeding points by increasing visibility of blood flow ( **Supplementary Fig. 1b** ) and to reduce frequency of switching observation modes during procedures by maintaining a color tone similar to that of WLI. To achieve this, in ACI mode, the spectral profile of the LED light source is controlled to make differences in shading of blood easier to observe, and changes in brightness and hue are limited to the red color range to display images in which blood flow is easily recognized ( **Supplementary Fig. 1c** ).

### Subjective evaluation

Nine endoscopic scenes captured with ACI and WLI were evaluated by eight endoscopists, four experts with experience with more than 100 gastric ESD cases, and four trainees with experience of fewer than 10 cases. Each endoscopic image of the same case was randomly presented on the left and right sides of one slide to conceal which IEE mode was used. Visibility of the dissection line and the submucosal vessel were determined via a 5-point Likert scale (left is superior, left is slightly superior, unchanged, right is slightly superior, right is superior). Each scale was converted to +2 points for ACI superior, +1 point for ACI slightly superior, 0 points for unchanged, -1 point for WLI slightly superior, and -2 points for WLI superior. Every image was evaluated using a 25-inch monitor with 1920 × 1080 resolution. Distribution and median of the points were examined. In addition, we calculated an intraclass correlation coefficient (ICC [2,1]), which is an indicator for evaluation of inter-evaluator reliability.

### Blue area comparison during ESD

Blue areas were extracted from three still endoscopic images taken during submucosal dissection using both ACI and WLI via the magic wand tool in the raster graphics editor “Photoshop” (Adobe Systems Co.). The size of the blue area in the submucosa was measured in pixels, and the size ratio of ACI to WLI (i.e., ACI/WLI) was calculated.

### RGB signal analysis of endoscopic images during ESD

Twelve regions of interest were set, four in each of the upper (mucosal side), middle (submucosal layer containing the injected blue solution), and lower (muscular side) layers. RGB signal intensity of every region was calculated using Photoshop. Average intensity in each red (R), green (G), and blue (B) signal was calculated for the upper, middle, and lower regions, both ACI and WLI. The average difference in each (R, G, B) intensity between ACI and WLI in the upper or lower region was compared with that in the middle region.

### Color and RGB signal analysis in ex vivo experiments

Indigo carmine was diluted two-fold from 1% (200 mL of 10% glycerol + 2 mL of indigo carmine) to 0.002% to create 10 blue solutions. After 1, 3, and 5 mL of each solution was added to a 12-well plate, endoscopic images of each diluted solution were acquired in ACI and WLI modes using the FUIJIFILM system or RDI (mode1) and WLI modes using a GIF-XZ1200 endoscope and EVIS X1 endoscopy system (Olympus Medical Systems Corporation, Tokyo, Japan) at a constant distance and light intensity. Eight endoscopists evaluated the dilution ratio at which the two shades could be distinguished as different to investigate the blue discrimination limit for ACI and WLI. Next, RGB signals in the endoscopic images of each concentration were calculated and the relationship between the blue discrimination limit and signal intensities was examined in both ACI and WLI. Finally, brightness of the ACI and RDI of each concentration was evaluated.

### Statistical analysis

Differences in each signal intensity (R, G, B) between the ACI and WLI in each region were compared with other regions via an unpaired *t* -test. *P* < 0.05 was considered to indicate statistical significance. All analyses, including ICC (2,1), were performed via SPSS Statistics 24 (SPSS Inc., Chicago, Illinois, United States).

## Results

### Visibility of dissection line in ACI vs WLI

**Fig. 2:**
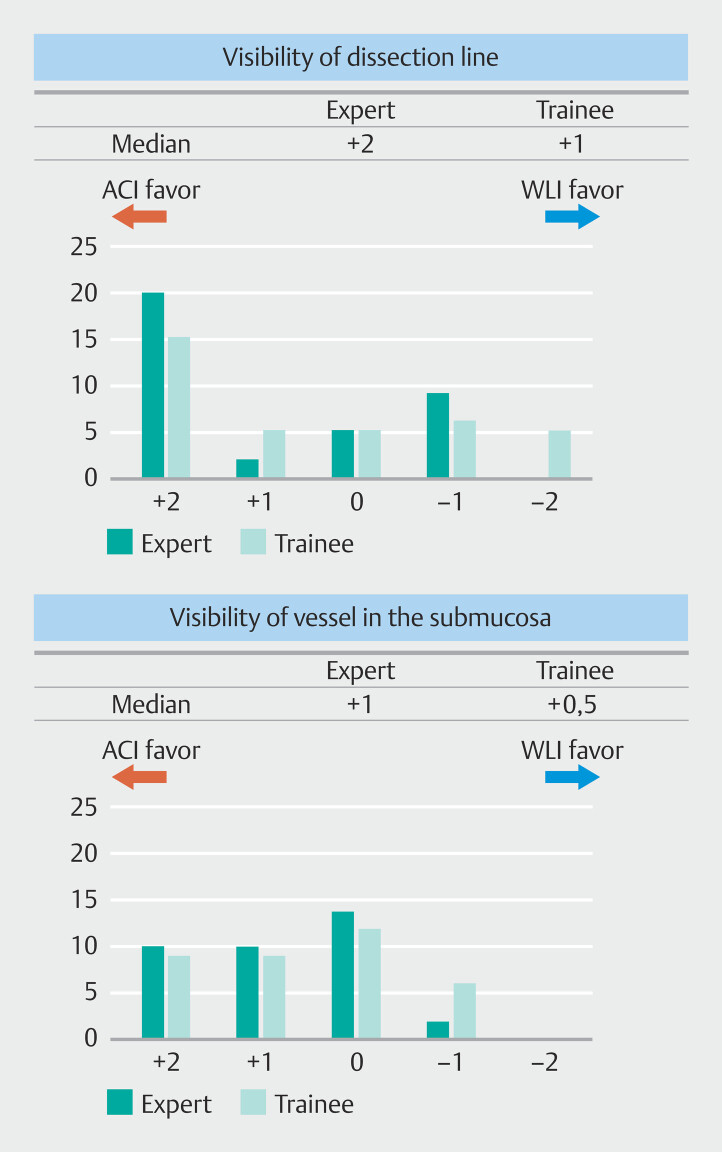
Median and distribution of Likert scores regarding visibility of the dissection line or vessels in the submucosa between ACI and WLI.

Median Likert scale score for visibility of the dissection line was +2 points (ACI is superior) for the experts and +1 point (ACI is slightly superior) for the trainees. A score of +2 points accounted for 48.1% (35/72) of the total ( Fig. 2 ).

The ICC (2,1) was 0.50 in experts and 0.54 in trainees. Median Likert scale score for the visibility of the submucosal vessels was +1 point for the experts and +0.5 points for the trainees. A score of 0 points (unchanged) was the most common at 36.1% (26/72), but the combined number of +2 and +1 points accounted for 50% (36/72) of the total. The ICC (2,1) was 0.37 in experts and 0.23 in trainees.

### Submucosal blue area in ACI

**Fig. 3:**
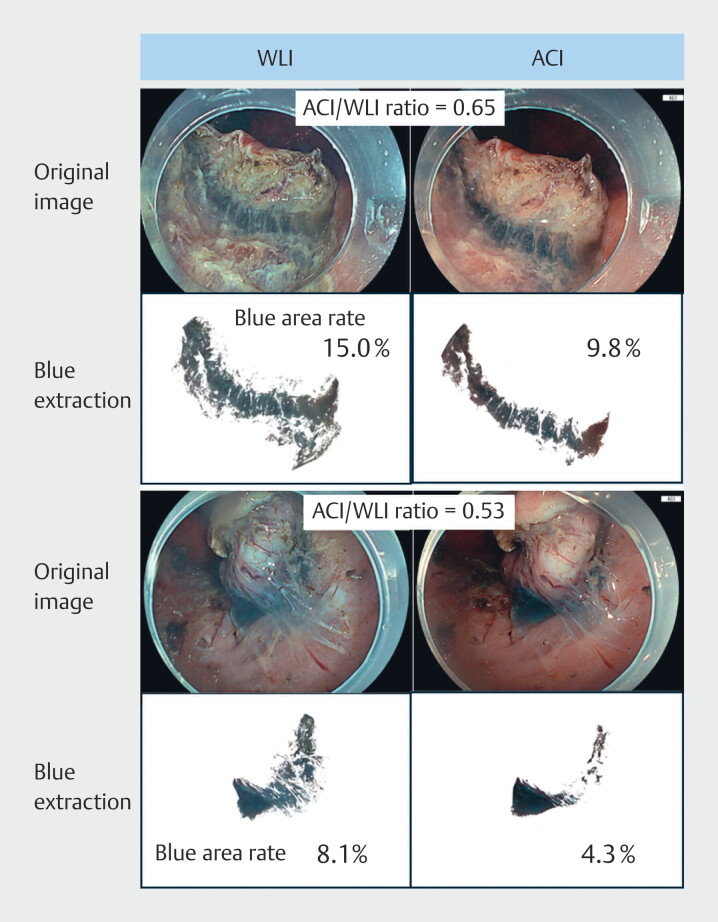
Representative image analyses of the ACI/WLI ratio of the blue area.

Compared with the blue area extracted from the three still endoscopic images of ESD, the ACI/WLI ratio of the blue area ranged from 0.53 to 0.65 ( Fig. 3 , **Supplementary Fig. 2** ), indicating that blue bandwidth was narrower in the ACI.

### Difference in red signal intensity between the submucosa and the surrounding area in ACI vs WLI

**Fig. 4:**
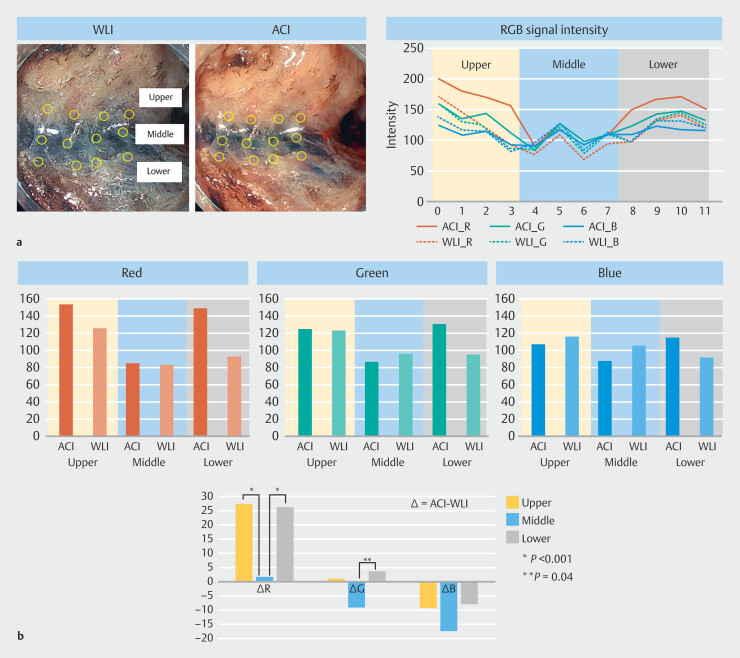
**a** Representative RGB signal intensity analysis of endoscopic images during submucosal dissection. The yellow circle in the endoscopic image indicates the region of interest in the upper (mucosal side), middle (submucosal side) and lower (muscular side) regions. **b** Average red, green and blue signal intensities for ACI and WLI in each region (upper). The difference between ACI and WLI in the RGB signal in each region (lower).

In the RGB signal intensity analysis of three endoscopic images during dissection, red, green, and blue signal intensities were lower in the middle region (submucosa) in both ACI and WLI ( Fig. 4 **a** , **Supplementary Fig. 3** ). Regarding average differences between ACI and WLI in the three endoscopic images, red signal intensity was significantly lower in the submucosa (middle region) than in the dissected mucosa (upper region) and muscular side (lower region) ( Fig. 4 **b** ). On the other hand, the difference in the green or blue signal between ACI and WLI was smaller than that of the red signal.

### Recognition of blue at low concentrations in ACI vs WLI

**Fig. 5:**
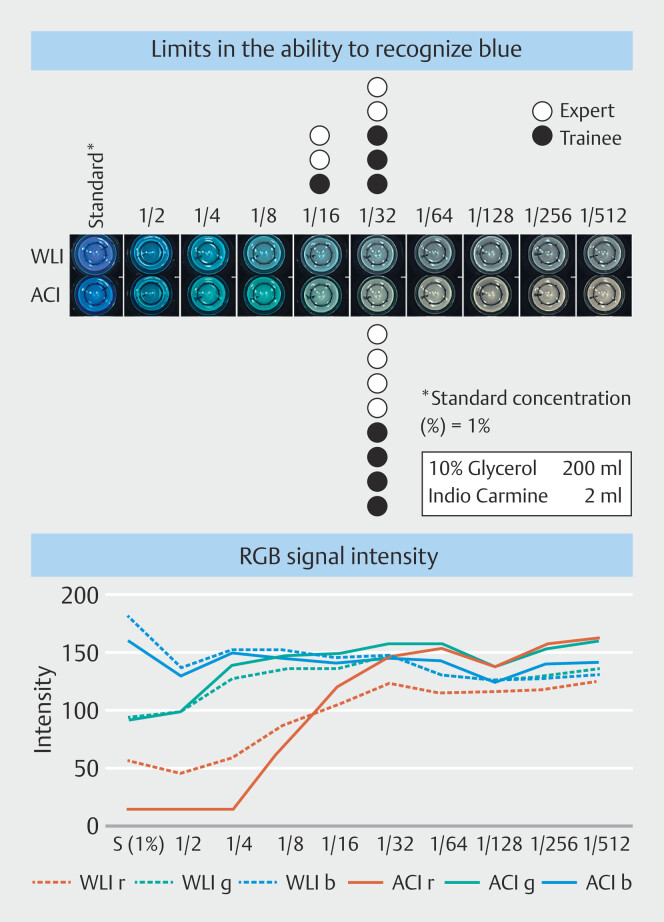
Endoscopic images of diluted solutions in 12-well dishes (from 1% to 1/512 dilution) acquired with ACI and WLI. The number of black or white circles indicates the limit of each endoscopist's ability to recognize blue (upper). RGB signal intensity of each dye dilution (lower)

In ACI, all eight evaluators experienced limits in their ability to recognize blue at a 1/16 dilution (0.06%). However, five of eight evaluators reported limits in their ability to recognize blue at a 1/32 dilution (0.03%) in WLI ( Fig. 5 ).

In the RGB signal intensity analysis for each concentration of indigo carmine-containing solution of 3 mL, green signal increased slightly as the dilution proceeded, whereas the blue signal hardly changed. These signal intensities were very similar between ACI and WLI. On the other hand, the red signal intensity increased as dilution proceeded, which was more marked in ACI. Red signal intensity differed between ACI and WLI depending on dilution. Specifically, red signal intensity in WLI was greater than that in ACI at a 1/4 dilution (0.25%), WLI and ACI were equivalent at 1/8 dilution (0.13%), and ACI was greater than WLI at 1/16 dilution (0.06%), indicating a shift in the red signal trend from the 1/4 dilution.

In this experiment, we found that the red signal in ACI was lower in the dark blue area and higher in the light blue area than in WLI. Notably, the blue discrimination limit concentration at the 1/16 dilution (0.06%) was consistent with the concentration at which the red signal in ACI overtook that in WLI.

Regarding the amount of indigo carmine diluted solution of 1, 3, and 5 mL, RGB signal analysis in only ACI revealed a big difference in the relationship between the red signal and green, or blue signal, at concentrations ranging from 1/2 to 1/4 between 1 ml and 3 or 5 mL ( **Supplementary Fig. 4** ).

RGB signal analysis of RDI and WLI revealed that the red signal in RDI was lower than the green or blue signals at all concentrations, which differed from that in WLI ( **Supplementary Fig. 5** ). Furthermore, L*, which indicates brightness in the L*a*b* color space, was lower in RDI than in ACI at an indigo carmine concentration of 0.25% or higher.

## Discussion

In ACI, a form of novel IEE, the dissection line of the submucosal layer during ESD is more reliable, and visibility of blood vessels in the submucosa is slightly improved compared with that of conventional WLI. The blue submucosal area, where an indigo carmine-containing solution was injected, was narrower in ACI than in WLI. In the RGB signal analyses of endoscopic images during ESD, red signal intensity was greater in ACI than in WLI on the mucosal and muscular layer sides near the submucosa. RGB signal analysis of ex vivo experiments also revealed that characteristics of the red signal differed between ACI and WLI. In a dark blue solution containing more than 0.13% indigo carmine, red signal intensity was similar or lower in ACI than in WLI. In contrast, in a more diluted blue solution, the red signal was greater in ACI than in WLI, making it difficult to recognize as a blue area. In other words, some diluted blue appears blue in WLI but not blue in ACI.

ESD is possible even with injection of a transparent solution. However, the dissection line is challenging to recognize, which can cause complications such as muscle layer injury. Therefore, local injection of a blue solution is essential to facilitate recognition of the appropriate dissection line 17 . If some blue solution injected into the submucosa leaks into the mucosal or muscular layer side, the blue areas are not always optimal dissection lines. Thus, there is sometimes confusion about which line should be dissected. In the ex vivo experiment, a solution containing 0.06% indigo carmine was the limit of blue recognition in ACI. At this concentration, red signal intensity of ACI was greater than that of WLI, making it difficult to recognize as blue. Based on RGB signal analysis of three kinds of volumes in Supplementary Fig. 4, a significant difference was observed at concentrations ranging from 1/2 to 1/4 between 1 and 3 mL or 5 mL. This would support the phenomenon that a small amount of solution appears white, whereas a larger amount tends to appear blue in ACI. From these data, it can be inferred that the recommended concentration of indigo carmine is between 0.25% and 0.5%. When this result is applied to an endoscopic image, the mucosal or muscular layer side where the blue local injection has leaked out corresponds with a low concentration of indigo carmine. We determined that these areas would be displayed on the monitor as not blue in ACI. In contrast, the submucosa with sufficient blue solution appeared blue because the red signal was equally low in both ACI and WLI. These results indicate that the blue submucosal area is narrower in ACI than in WLI. Generally, fibrosis in the submucosa has a limited injection space, and a narrow dissection line makes ESD challenging. However, the narrower blue submucosal area in ACI than in WLI after a sufficient injection differs from the narrowed space due to fibrosis resulting from less injection. In other words, a narrow line means an appropriate line without leaking extra light blue. Performing the entire ESD in ACI, therefore, would improve recognition of the dissection line because only submucosal areas contain sufficient injected dye to be displayed in blue, and bleeding points are more visible. This approach contributes to reducing complications such as muscle layer injury and perforation during ESD.

RDI, developed before ACI, reportedly results in better visualization of bleeding points, which shortens hemostasis during ESD in the esophagus and colon 14 . Meanwhile, one report shows that RDI improves visibility of the muscle layer demarcation line in colorectal ESD 11 . To the best of our knowledge, there are only two case reports 18 19 and one retrospective study about ESD using RDI alone 20 . In that study, dissection in the submucosal layer was significantly faster even though procedure time was not different. Moreover, one perforation occurred in 25 cases with RDI (0 in 57 cases with WLI), although the difference was not significant. In the current study, RGB signal analysis of RDI showed that the red signal was consistently lower than green or blue signals at all concentrations, resulting in a blue-based image, even without injection of indigo carmine. Furthermore, the brightness (L*) was lower in RDI than in ACI, suggesting that the indigo carmine-injected submucosal image was darker, which is consistent with our feelings. To our understanding, the utility of RDI for full-time ESD is still controversial, and its use is rare. A switch from WLI to RDI is usually performed for hemostasis when bleeding occurs. On the other hand, there are two ESD case reports concerning ACI. We previously reported that ACI changed only the red color range and maintained other color tones similar to those of WLI in gastric ESD 16 . Another study reported that ACI enhances visibility of vessels in the submucosa in colorectal ESD, which makes ESD easier and safer 21 . This study has several limitations. First, it was a retrospective study at a single facility with a small number of cases. Second, we could not wholly exclude potential bias because the evaluators could identify ACI images from the color tone. Third, selection bias of the endoscopic case would not be entirely excluded, although we selected every pair of images with the same case. Fourth, visibility of the dissection line and vessels is subjective and clinical utility of ESD is speculative. Fifth, this study did not directly compare the procedure time or complication rate between ACI and WLI. A prospective, randomized, multi-institutional study is needed to verify efficacy of using ACI for the entire ESD procedure.

## Conclusions

In conclusion, ACI emphasizes the submucosa as blue only where sufficient solution is injected, which would support the recognition of the dissection line during ESD.

## Supplementary Material

Supplementary Material Zusatzmaterial
