# Supplementary material for: Amber-red color imaging makes the dissection line more evident during gastric endoscopic submucosal dissection
Source: Endosc Int Open. 2025 Sep 15;13:a26947445. doi: 10.1055/a-2694-7445 (PMC12445331; doi:10.1055/a-2694-7445)

Supplementary Fig. 1 a Illumination of the spectrum in WLI and ACI.

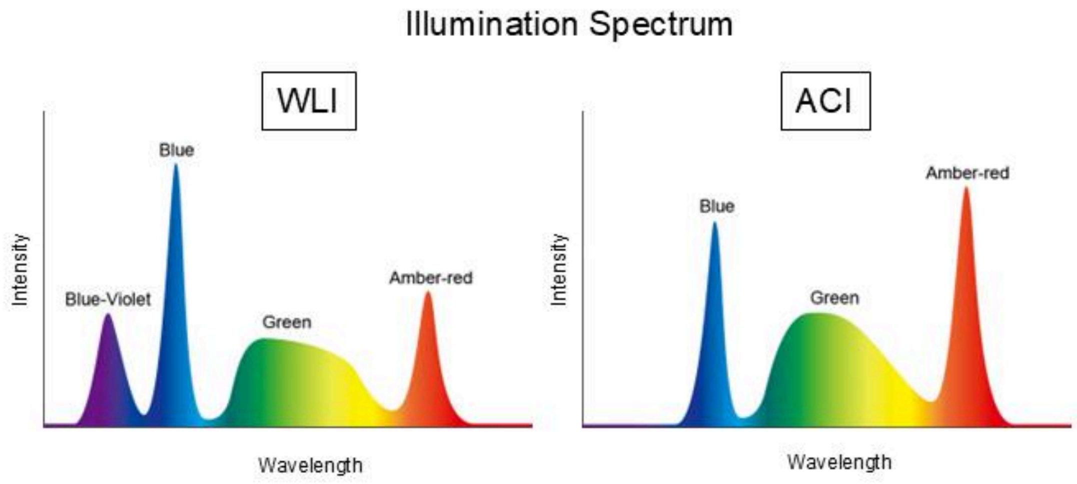

Supplementary Fig. 1b Representative endoscopic image of a bleeding scene in WLI and ACI.

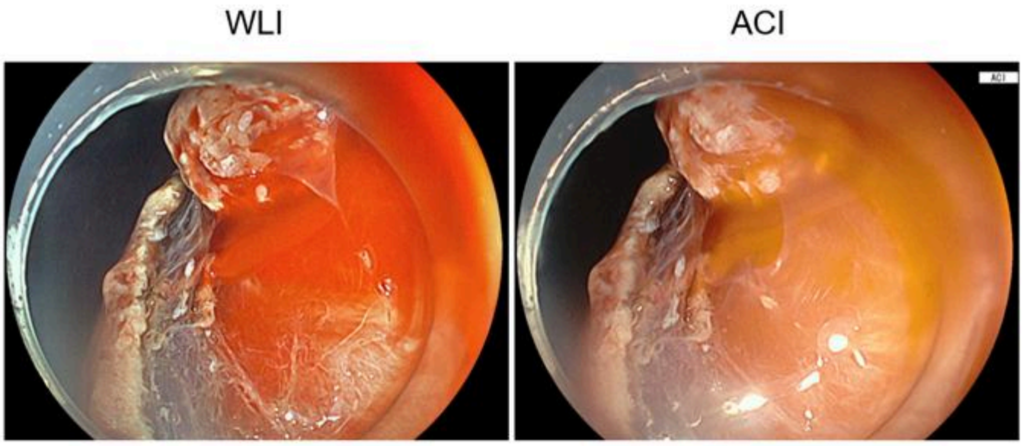

Supplementary Fig. 1c Conceptual diagram of the image enhancement algorithm in ACI.

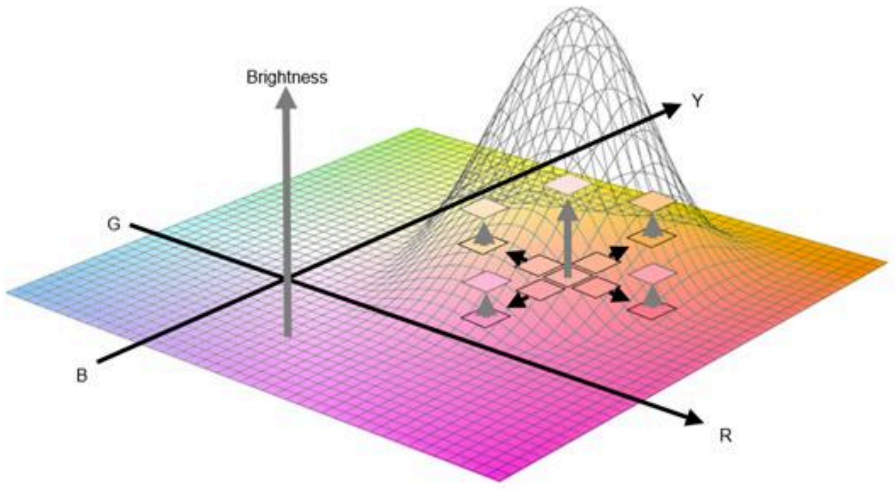

Supplementary Fig. 2 Image analysis of the ACI/WLI ratio of the blue area.

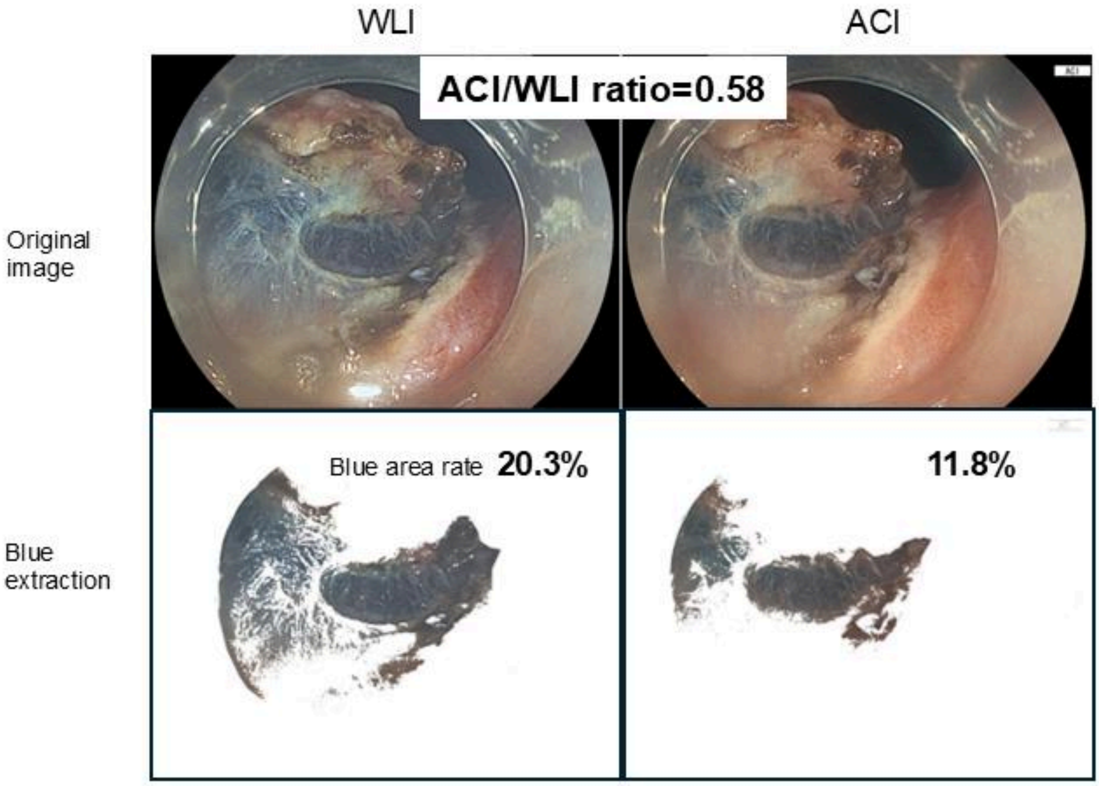

Supplementary Fig. 3 RGB signal intensity analyses of endoscopic images during submucosal dissection.

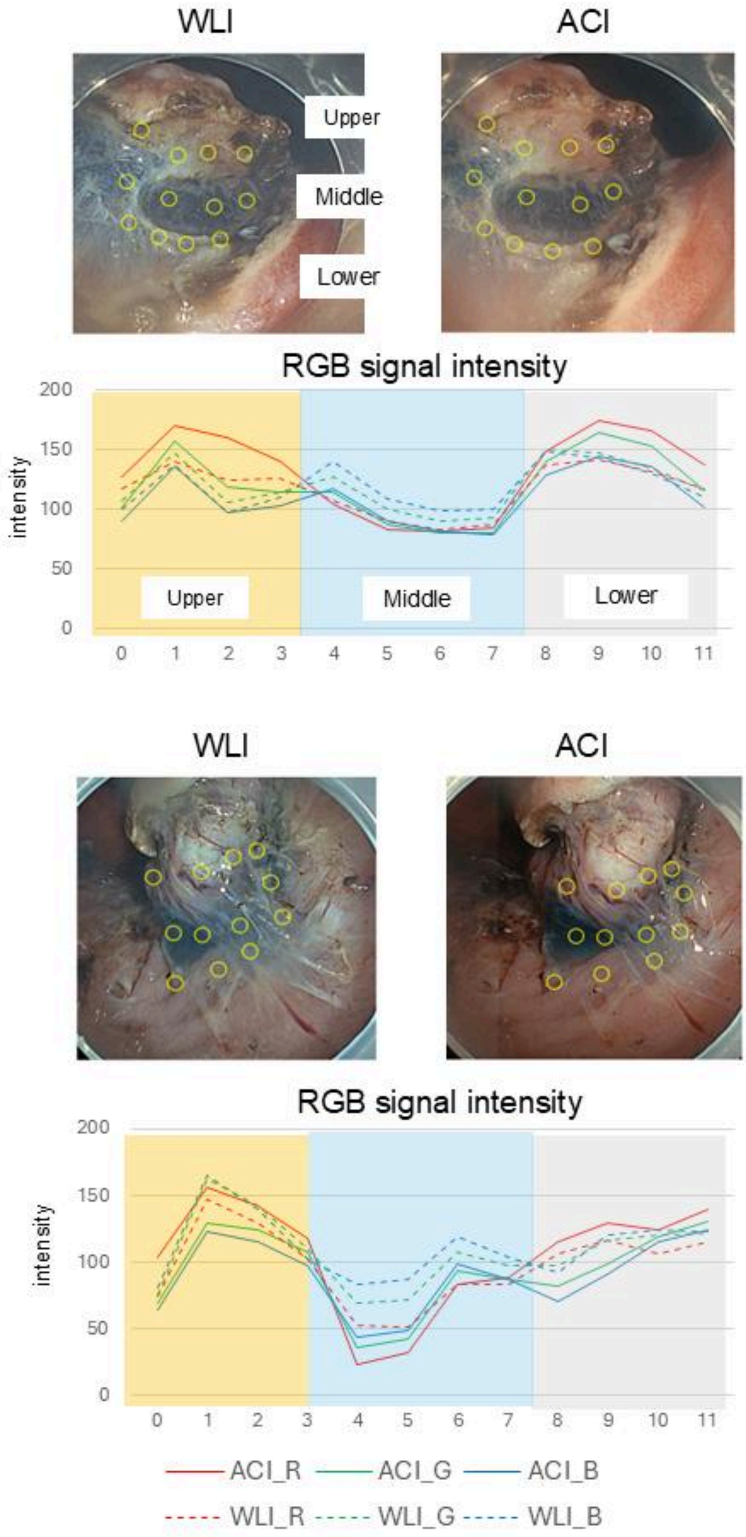

Supplementary Fig. 4 RBG signal intensity analysis in ACI with varying amounts of indigo carmine diluted solutions: 1, 3, and 5 mL.

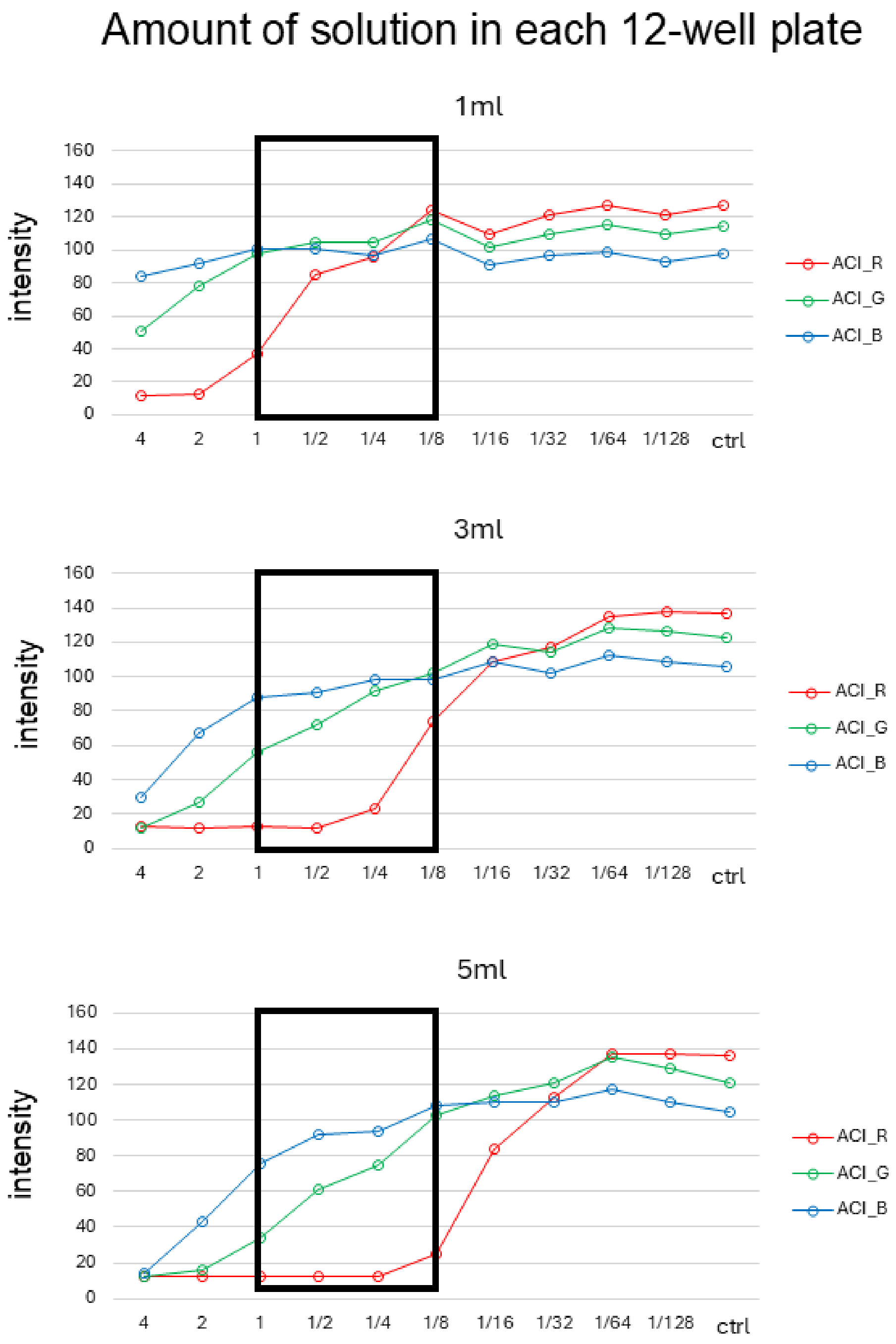

Supplementary Fig. 5 Representative image of each dye solution in WLI and RDI (higher) RGB signal intensity of each dye dilution between WLI and RDI (middle).
The L* of L*a*b* color space of each dye solution in ACI and RDI (lower).

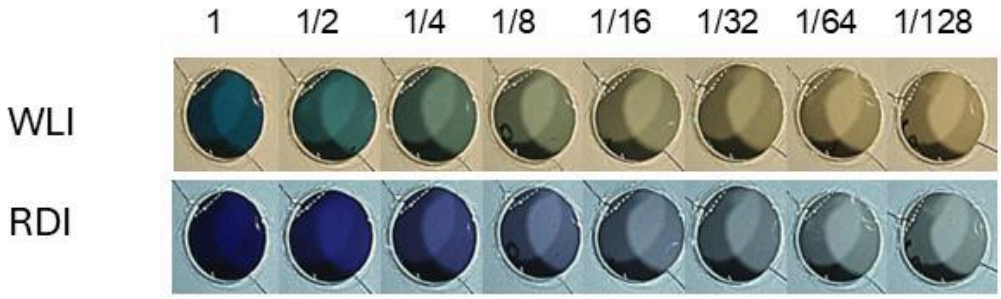

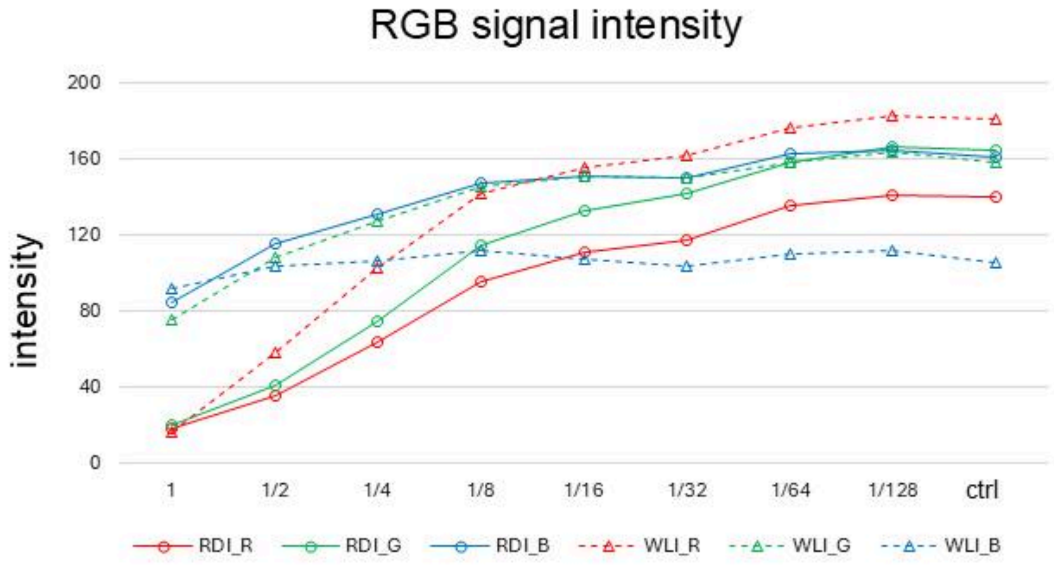

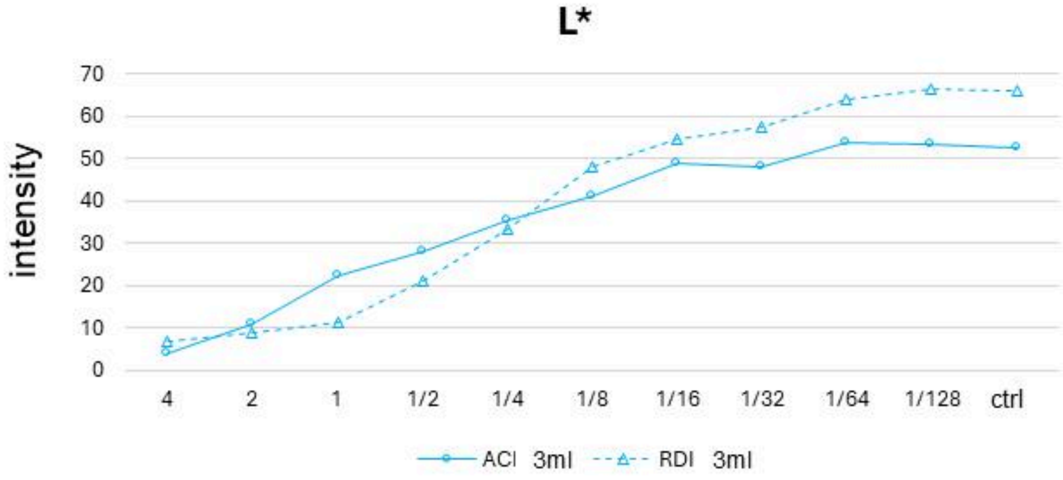

Supplement: Supplementary file 1 — Supplementary Material [file 10-1055-a-2694-7445_26957243.pdf]
